# MAGI-1 Interacts with Nephrin to Maintain Slit Diaphragm Structure through Enhanced Rap1 Activation in Podocytes*[Fn FN2]

**DOI:** 10.1074/jbc.M116.745026

**Published:** 2016-10-05

**Authors:** Jie Ni, Sujin Bao, Ruth I. Johnson, Bingbing Zhu, Jianhua Li, Justin Vadaparampil, Christopher M. Smith, Kirk N Campbell, Florian Grahammer, Tobias B. Huber, John C. He, Vivette D. D'Agati, Andrew Chan, Lewis Kaufman

**Affiliations:** From the a Division of Nephrology, Icahn School of Medicine at Mount Sinai, New York, New York 10029,; the b Division of Nephrology, First Affiliated Hospital of Harbin Medical University, Harbin, China 150001,; the c Saint James School of Medicine, Saint Vincent and the Grenadines,; the d Biology Department, Wesleyan University, Middletown, Connecticut, 06459,; the e Department of Nephrology, Laboratory of Renal Disease, Putuo Hospital, Shanghai University of Traditional Chinese Medicine, Shanghai, China 200062,; the f Department of Medicine IV, Medical Center-University of Freiburg, Faculty of Medicine, University of Freiburg, 79106 Freiburg, Germany,; the g BIOSS Center for Biological Signaling Studies, Albert-Ludwigs-University Freiburg, 79104 Freiburg, Germany,; h FRIAS, Freiburg Institute for Advanced Studies and Center for Systems Biology (ZBSA), Albert-Ludwigs-University, 79104 Freiburg, Germany,; the i Department of Pathology, Columbia University Medical Center, New York, New York 10032, and; the j School of Biomedical Sciences, Chinese University of Hong Kong, Hong Kong, China

**Keywords:** mouse genetics, nephrology, podocyte, proteinuria, Ras-related protein 1 (Rap1)

## Abstract

MAGI-1 is a multidomain cytosolic scaffolding protein that in the kidney is specifically located at the podocyte slit diaphragm, a specialized junction that is universally injured in proteinuric diseases. There it interacts with several essential molecules, including nephrin and neph1, which are required for slit diaphragm formation and as an intracellular signaling hub. Here, we show that diminished MAGI-1 expression in cultured podocytes reduced nephrin and neph1 membrane localization and weakened tight junction integrity. Global *magi1* knock-out mice, however, demonstrated normal glomerular histology and function into adulthood. We hypothesized that a second mild but complementary genetic insult might induce glomerular disease susceptibility in these mice. To identify such a gene, we utilized the developing fly eye to test for functional complementation between MAGI and its binding partners. In this way, we identified diminished expression of fly Hibris (nephrin) or Roughest (neph1) as dramatically exacerbating the effects of MAGI depletion. Indeed, when these combinations were studied in mice, the addition of nephrin, but not neph1, heterozygosity to homozygous deletion of MAGI-1 resulted in spontaneous glomerulosclerosis. In cultured podocytes, MAGI-1 depletion reduced intercellular contact-induced Rap1 activation, a pathway critical for proper podocyte function. Similarly, *magi1* knock-out mice showed diminished glomerular Rap1 activation, an effect dramatically enhanced by concomitant nephrin haploinsufficiency. Finally, combined overexpression of MAGI-1 and nephrin increased Rap1 activation, but not when substituting a mutant MAGI-1 that cannot bind nephrin. We conclude that the interaction between nephrin and MAGI-1 regulates Rap1 activation in podocytes to maintain long term slit diaphragm structure.

## Introduction

Membrane-associated guanylate kinase 1 (MAGI-1) is a member of the MAGUK family of scaffolding proteins. It contains five PDZ domains, two WW domains, and a catalytically inactive guanylate kinase domain. MAGI-1 is expressed in most organs (1), but in kidney, it is specifically localized to the cytoplasmic side of the podocyte slit diaphragm (2). Via its multiple protein-interacting domains, MAGI-1 is known to interact with many other important podocyte proteins, including nephrin (2), synaptopodin (3), α-actinin-4 (3), and dendrin (4). There are three MAGI-1 splice variants (designated A, B, and C), each introducing a unique C-terminal sequence, but all three use the identical initiation ATG methionine (1). The MAGI subfamily also includes MAGI-2 and MAGI-3 that contain identical domain organizations. Three recent studies all demonstrate that MAGI-2 has a critical role in formation of podocyte foot processes and slit diaphragms (5–7). In fact, global *magi2* knock-out mice demonstrate early lethality caused by severe podocyte failure with anuria (5, 7).

Podocyte injury occurs in all proteinuric kidney diseases independent of the underlying cause, resulting in loss of foot processes and slit diaphragm architecture. Two key components of the slit diaphragm, nephrin and neph1, form cross-strand complexes that bridge and anchor the porous slit diaphragm structure. These two molecules, in an intricate complex of numerous cytoplasmic proteins that includes MAGI-1, also coordinate outside-in signaling events that link to the actin cytoskeleton (8). The essential roles of nephrin and neph1 are reflected in the severe phenotypes of loss of function mutations in these genes in mice (9, 10), but the importance of MAGI-1 as a component of this complex is completely unknown.

In addition to its role as a scaffolding protein, MAGI-1 also modulates several intracellular signaling networks, including pathways already known to be important in the podocyte injury response. For example, after cell-cell contact, MAGI-1 is required for activation of the small GTPase Rap1 (11), a critical mediator of integrin activation in podocytes (12). In fact, diminished Rap1 signaling in podocytes induces severe glomerular disease in mice and is associated with human glomerular disease pathogenesis (12). Multiple upstream pathways, including GTPase-activating proteins (GAPs)^3^ and guanine nucleotide exchange factors (GEFs), work in concert to maintain proper Rap1 balance both at baseline and during physiological stress (13). The role of MAGI proteins in potentially regulating Rap1 activation in podocytes, however, has not been reported previously.

In the current work, we find that under basal conditions, *magi1* knock-out mice have long term normal glomerular architecture and function. This suggests that loss of MAGI-1 alone, unlike MAGI-2, represents a relatively mild genetic insult that may be compensated for by other genes. However, as is often the case in human FSGS pathogenesis, we hypothesized that a second mild but complementary genetic insult might be able to induce podocyte dysfunction in our model. To identify such a gene, we studied pattern development of the compound eye of the fruit fly (14, 15), *Drosophila melanogaster*, an intricate process that relies on homologs of mammalian slit diaphragm proteins. We found that flies that were deficient in MAGI demonstrated only mild patterning defects that were made dramatically more severe by concomitant reduction of either Hibris or Roughest, homologues of nephrin and neph1, respectively. Similarly, in mice, the absence of MAGI-1 in combination with heterozygosity of nephrin, but not neph1, augmented the injury response substantially. The combination of these two relatively mild genetic insults induced glomerulosclerosis, suggesting long term genetic complementation. This interaction was also reflected on a functional level, where the combined loss of MAGI-1 and nephrin synergistically prevented Rap1 activation. Overall, our work establishes *MAGI-1* as a disease-modifying gene that probably plays an important role in podocyte remodeling in human glomerular diseases.

## Results

### 

#### 

##### Reduced MAGI-1 Expression Diminishes Membrane Nephrin and neph1

**FIGURE 1.**
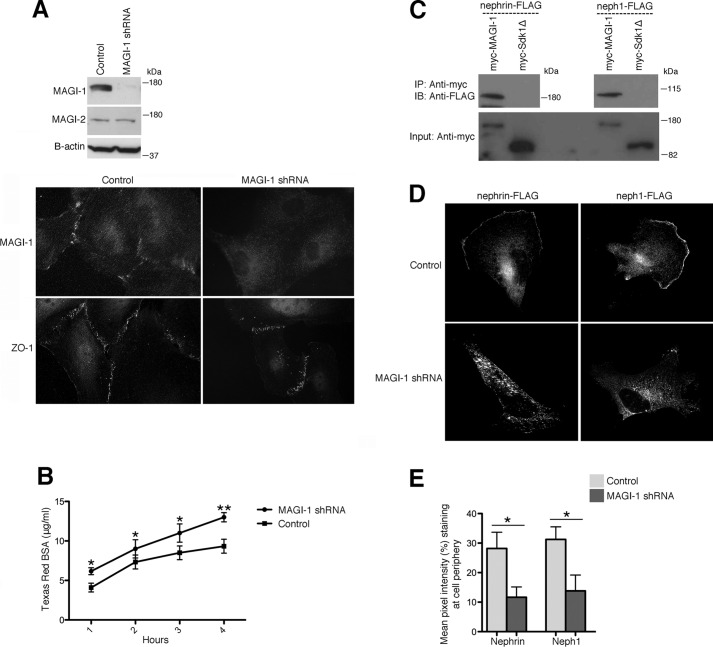
**Diminished MAGI-1 expression in podocytes weakens tight junctions and causes loss of membrane nephrin and neph1.** *A*, *magi1* knockdown podocytes show dramatic loss of MAGI-1, but not MAGI-2, protein expression by Western blotting and immunocytochemistry compared with control transduced cells. *B*, confluent *magi1* knockdown podocytes demonstrate a substantial increase in paracellular albumin flux compared with control cells. *, *p* < 0.02; **, *p* < 0.01. *C*, both FLAG-tagged nephrin and neph1 co-immunoprecipitate with Myc-MAGI-1 but not with Myc-sdk1Δ from doubly transfected 293T cellular lysates. *D*, *magi1* knockdown podocytes that are transfected with either FLAG-tagged nephrin or neph1 show relative loss of membrane FLAG expression compared with control cells. *E*, quantification of membrane FLAG expression was performed. Mean pixel intensity of membrane FLAG was calculated by averaging three different experiments (*n* = 20 randomly selected cells from each group for each experiment). *IP*, immunoprecipitation; *IB*, immunoblotting. *, *p* < 0.015. *Error bars*, S.E.

Using lentiviral transduction of a conditionally immortalized human podocyte cell line, we generated stable *magi1* knockdown podocytes (Fig. 1*A*, *top*). MAGI-2 expression levels were similar between the knockdown and control transduced cell lines, confirming both the specificity of the MAGI-1 shRNA and the absence of any compensatory increases in MAGI-2. By immunofluorescence, control transduced podocytes expressed MAGI-1 specifically at intercellular junctions, whereas *magi1* knockdown podocytes lacked significant MAGI-1 expression (Fig. 1*A*, *bottom*). Robust expression of the tight junction protein ZO-1 was present in both cell lines. To evaluate the effect of MAGI-1 loss on tight junction integrity, we performed albumin permeability experiments comparing confluent *magi1* knockdown podocytes and controls (Fig. 1*B*). *magi1* knockdown podocyte monolayers allowed increased passage of fluorescently labeled albumin over time, implying less robust tight junction formation in these cells. Although the direct interaction of MAGI-1 with nephrin has been well established (2), an interaction with neph1 has not been described previously. We performed co-immunoprecipitation experiments using Myc-MAGI-1 as bait to pull down FLAG-tagged nephrin, neph1, and a sidekick-1 truncation mutant (sdk-1Δ) (Fig. 1*C*). sdk-1Δ lacks its PDZ binding domain and is unable to mediate a direct interaction with MAGI-1 (16), making it an appropriate negative control for these studies. Protein-protein interactions were seen between MAGI-1 and both nephrin and neph1, but not with sdk-1Δ. To test whether MAGI-1 scaffolding facilitated nephrin and neph1 subcellular localization, we expressed FLAG-tagged nephrin and neph1 in control and *magi1* knockdown podocytes and performed immunofluorescence with an anti-FLAG antibody (Fig. 1*D*). Whereas control podocytes showed significant nephrin and neph1 expression at the cell membrane and cell periphery, loss of MAGI-1 dramatically reduced this membrane staining with an apparent increase in cytoplasmic reactivity. A quantitative assessment of these images confirmed these findings (Fig. 1*E*).

##### Global magi1 Knock-out Mice Do Not Have Overt Glomerular Dysfunction at Baseline

**FIGURE 2.**
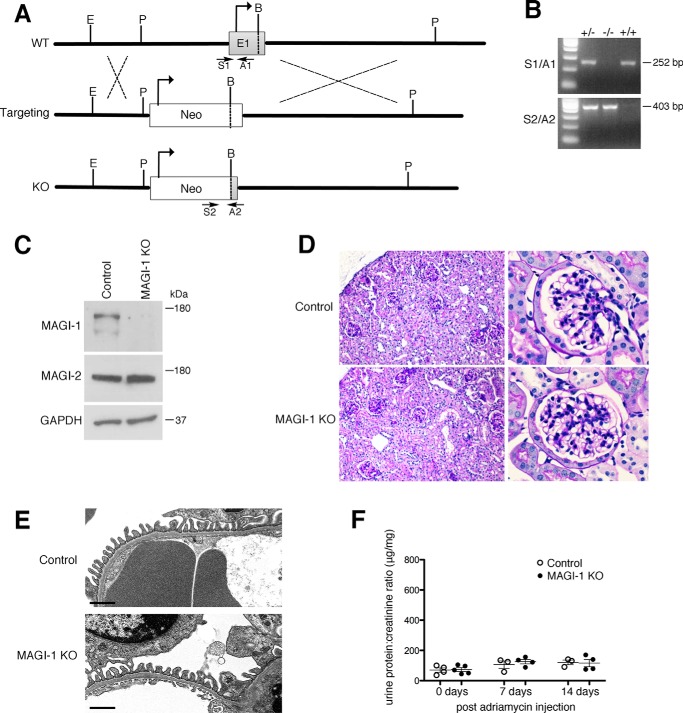
***magi1* knock-out mice have normal glomerular histology at baseline.** *A*, schematic diagram of targeting strategy for generation of *magi1* KO mouse line (*E*, EcoRI; *P*, PstI; *B*, BsiWI; *TV*, targeting vector). *B*, genotyping using allele-specific PCR strategy (*S1/A1*, WT-specific primer pair, product size 252 bp); *S2/A2*, KO-specific primer pair, product size 403 bp). *C*, characterization of *magi1* KO mice by Western blotting for MAGI-1 and MAGI-2 on kidney cortex. *D*, PAS staining of *magi1* KO and control mice demonstrates normal glomerular histology. *E*, electron microscopic images show normal foot processes in *magi1* KO mice similar to littermate controls. *Scale bars*, 1 μm. *F*, *magi1* KO mice (*n* = 5) did not show increased susceptibility to adriamycin nephropathy compared with WT controls (*n* = 4). One mouse per group died within 1 day of adriamycin administration. *Error bars*, S.E.

To better understand the role of MAGI-1 in podocyte function, we generated constitutive *magi1* knock-out mice. MAGI-1 is expressed as three unique splice isoforms that all use the identical ATG start codon. To affect all variants, we developed a targeting strategy that included replacing 3.2 kb of MAGI-1 genomic DNA, including most of exon 1 together with its ATG start codon as well as its upstream promoter region (Fig. 2*A*). A PCR-based approach using genomic DNA was used to detect the presence or absence of the wild type and knock-out alleles (Fig. 2*B*). Western blotting of the kidney cortex confirmed complete loss of MAGI-1 protein expression in the null mice without significant changes in MAGI-2 expression (Fig. 2*C*). *magi1* null mice did not show glomerular pathological changes by either light (Fig. 2*D*) or electron microscopic analysis (Fig. 2*E*). No significant histological changes or proteinuria were detected in *magi1* null mice up to an age of 2 years. Furthermore, 8-week-old male *magi1* knock-out mice, on a resistant C57Bl/6 genetic background, failed to develop susceptibility to adriamycin nephropathy compared with control mice (Fig. 2*F*).

##### MAGI Interacts with Nephrin and neph1 in the Drosophila Pupal Eye

**FIGURE 3.**
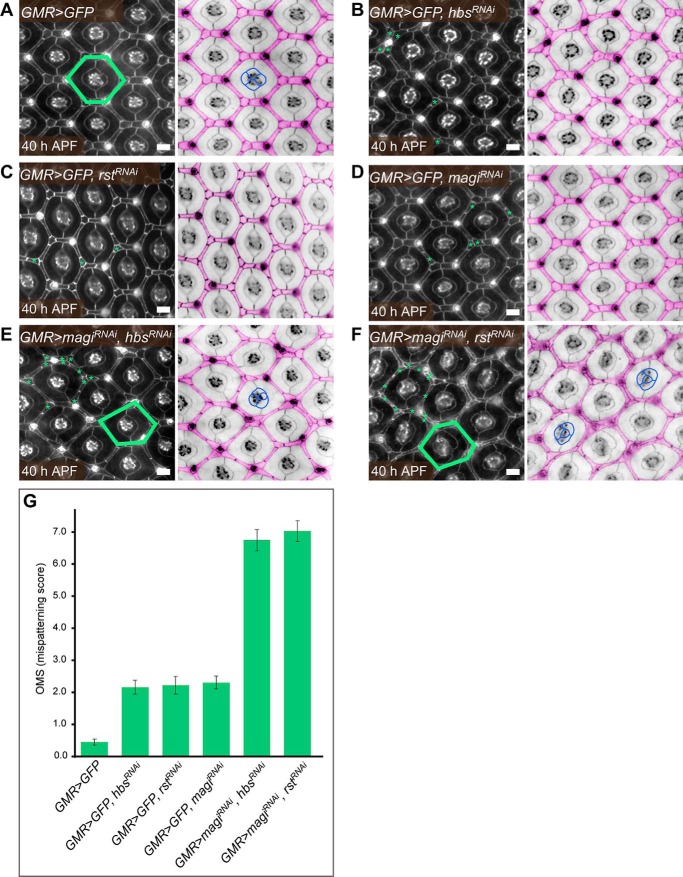
***magi*, *hbs*, and *rst* interact to correctly pattern the *Drosophila* pupal eye.** *A*, cells were arranged in a wild type patterning when *GFP* was expressed in the *Drosophila* pupal eye. In the *right-hand panel*, the interommatidial cells are *pseudocolored* in *pink*. These arranged into stereotypical shapes and positions to generate a hexagonal lattice. One hexagon is indicated in *green* (*left*). In the *center*, four cone cells arranged into a stereotypical pattern (example *outlined* in *blue*). The hexagonal pattern of the eye was mildly disrupted when *hbs* (*B*), *rst* (*C*), or *magi* (*D*) was modestly reduced by expression of RNAi transgenes under the control of GMR-Gal4. *hbs* and *rst* are fly homologs of mammalian nephrin and neph1, respectively, whereas *magi* is equivalent to mammalian MAGI-1 or MAGI-2. In each of these genotypes, *GFP* was co-expressed with the RNAi transgenes. Examples of incorrectly positioned or shaped cells are indicated with *green asterisks*. Co-expression of *magi^RNAi^* and either *hbs^RNAi^* (*E*) or *rst^RNAi^* (*F*) greatly disrupted patterning of the eye. Examples of incorrectly shaped or positioned cells about one ommatidium (a unit eye) are indicated in each *left-hand panel*. Numerous excess cells were observed. These errors frequently distorted the hexagonal lattice (examples indicated in *green*). Misarrangements of the four central cone cells were frequent (examples indicated in *blue*). *Scale bars*, 10 μm. All eyes were dissected at 40 h after puparium formation (*APF*). Apical adherens junctions were visualized with an antibody to E-cadherin. *G*, quantification of the mean number of patterning errors per ommatidium observed in each genotype. *Error bars*, S.E. See supplemental Table 1 for full statistical analysis.

We hypothesized that a second mild but complementary genetic insult might expose a glomerular phenotype in *magi1* null mice. To look for such complementary genes, we utilized the developing *Drosophila* eye to test for genetic interaction between MAGI (*magi*) and its interacting proteins Hibris (*hbs*) and Roughest (*rst*), fly homologs of vertebrate nephrin and neph1, respectively. In this system, interommatidial cells (IOCs) rearrange to form a highly organized repetitive hexagonal lattice, an elaborate process that depends on the careful regulation of cell-cell adhesion by *Drosophila* homologs of mammalian slit diaphragm proteins, such as *hbs* and *rst* (14, 15). We utilized the GMR-GAL4/UAS system to drive expression of specific RNAi and GFP transgenes in the developing pupal eye, resulting in modest reductions in gene expression. The IOCs arranged into a wild type hexagonal lattice when GFP alone was expressed (Fig. 3*A*). This hexagonal pattern was only slightly disrupted when *hbs*, *rst*, or *magi* RNAi alone was expressed (Fig. 3, *B–D*). Mild defects included incorrect positioning or shape of IOCs. When *magi* RNAi was co-expressed with either *hbs* or *rst* RNAi, however, severe patterning defects were evident, including numerous excess IOCs with frequently disturbed cell shape and positioning (Fig. 3, *E* and *F*). These defects often distorted the usual hexagonal lattice and induced misplacement of the four central cone cells. A quantitative analysis of eye defect severity confirmed similar levels of mispatterning between *magi*/*hbs* and *magi*/*rst* RNAi combinations (Fig. 3*G* and supplemental Table 1).

##### Nephrin, but Not neph1, Haploinsufficiency Induces FSGS in magi1 Knock-out Mice

**FIGURE 4.**
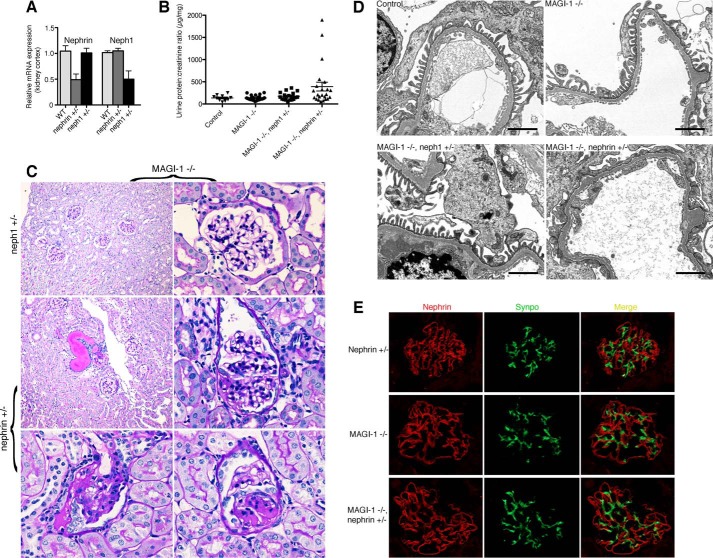
**Nephrin, but not neph1, haploinsufficiency in *magi1* knock-out mice induces late onset FSGS.** *A*, *Nephrin* and *Neph1* heterozygous mice show ∼50% reduction in mRNA expression of nephrin and neph1, respectively. *B*, several *magi1* KO mice that are also heterozygous for nephrin (*n* = 4 of 23 mice), develop significant proteinuria at an age of 12 months as compared with *magi1* KO alone (*n* = 0 of 25), KO mice that are also heterozygous for neph1 (*n* = 0 of 20), or control mice (*n* = 0 of 10). All mice were ∼12 months of age at the time of urine collection. *C*, PAS staining of kidneys from proteinuric mice shows tubular dilatation with cast formation (*middle panel*, *left*) associated with both focal (*middle panel*, *right*) and global glomerulosclerosis (*bottom panels*). Kidneys from MAGI-1 KO mice that were also heterozygous for neph1 revealed normal appearing histology (*top panels*). *D*, electron microscopy images reveal areas of diffuse podocyte foot process effacement in *magi1* KO mice that are also heterozygous for nephrin (*bottom right panel*) but normal podocyte architecture in the other groups. *Scale bar*, 2 μm. *E*, double immunofluorescent staining for nephrin and synaptopodin failed to show significant differences in nephrin localization between groups. *Error bars*, S.E.

Based on these *Drosophila* genetic interaction studies, we hypothesized that partial loss of nephrin or neph1 might enhance the phenotype of *magi1* knock-out mice. To test this, we generated *magi1* null mice that were also heterozygous for either nephrin or neph1. By quantitative PCR performed on kidney cortex, nephrin and neph1 heterozygous mice demonstrated ∼50% reduction in nephrin and neph1 mRNA, respectively (Fig. 4*A*). At approximately 1 year of age, the combination of complete loss of MAGI-1 with partial loss of nephrin, but not neph1, resulted in overt proteinuria in some mice (Fig. 4*B*). Histological analysis revealed FSGS in the *magi1* knock-out mice that were also heterozygous for nephrin (4 of 23 mice), but not for neph1 (0 of 20 mice) or MAGI-1 alone (0 of 25 mice) (Fig. 4*C*). FSGS lesions were present in 2–8% of glomeruli in affected mice. FSGS was of variable intensity, ranging from small focal lesions with characteristic synechia formation to more globally appearing lesions. Affected glomeruli showed podocyte swelling as well as apparent podocyte loss with overlying areas of proliferative glomerular epithelial cell activation. Electron microscopy demonstrated severe podocyte effacement in affected glomeruli but normal slit diaphragm structure in the other groups (Fig. 4*D*).

Our podocyte culture data (Fig. 1, *D* and *E*) had suggested that loss of MAGI-1 might result in slit diaphragm instability by causing mislocalized nephrin expression. However, detailed double labeling immunofluorescent experiments failed to detect any significant alteration in nephrin localization in proteinuric mice compared with *magi1* knock-out or nephrin heterozygous littermates (Fig. 4*E*). This suggested that an alternative mechanism was primarily responsible for podocyte dysfunction in our model.

##### MAGI-1 Enhances Rap1 Signaling and Is Facilitated by Nephrin

**FIGURE 5.**
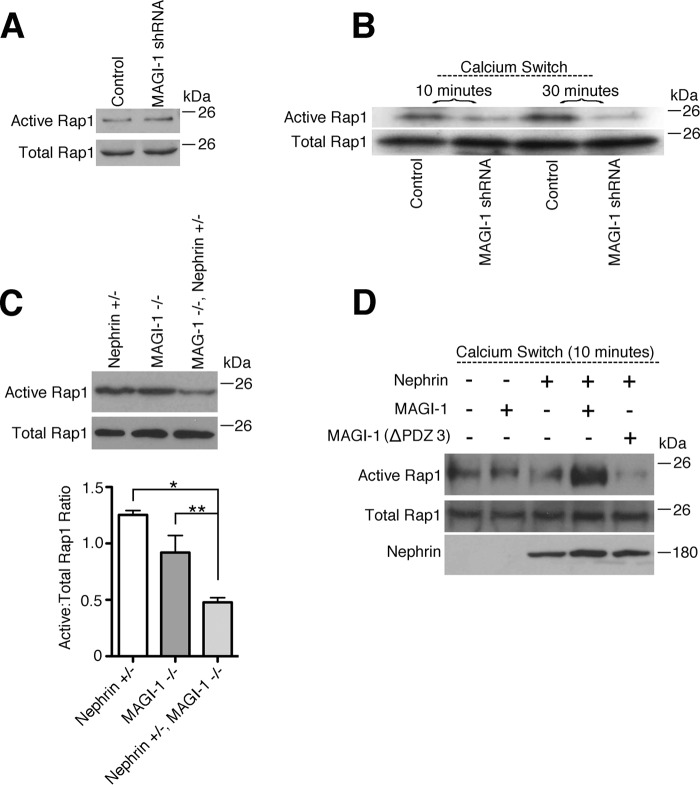
**Nephrin and MAGI-1 interact to augment podocyte Rap1 activation.** *A*, under the basal condition, levels of active Rap1 are similar between control and MAGI-1 knockdown podocytes. *B*, confluent control and *magi1* knockdown podocytes had intercellular contacts disrupted by Ca^2+^ chelation and then restored by the addition of full Ca^2+^-containing medium (calcium switch). Cellular lysates at the time points indicated after calcium switch were assayed for levels of active Rap1. *magi1* knockdown podocytes were resistant to Rap1 activation in this setting. *C*, glomerular lysates from two mice per group were pooled and then assayed for levels of active Rap1. *magi1* KO mice that were also heterozygous for nephrin showed exaggerated loss of glomerular Rap1 activation. The experiment was repeated three times (three sets of two mice per group, 18 mice total), and a representative image is shown (*top*). Band intensities for all blots were calculated, and the composite ratios between active and total Rap1 relative intensities were computed (*bottom*). *, *p* = 0.0002; **, *p* < 0.05. *D*, confluent human podocytes were transfected with the corresponding expression plasmid(s) and then exposed to Ca^2+^ switch. After 10 min, cellular lysates were collected and assayed for levels of active Rap1. Augmented levels of Rap1 activation were evident when both full-length MAGI-1 and nephrin were co-expressed. This effect was lost when substituting a mutant MAGI-1 that is missing its nephrin-interacting domain. *Error bars*, S.E.

MAGI-1 is a known upstream activator of Rap1 signaling (11), a pathway whose dysregulation results in severe podocyte dysfunction (12). To investigate whether MAGI-1 regulates Rap1 activation in podocytes, we compared levels of active Rap1 (Rap1-GTP) in *magi1* knockdown and control podocytes. At baseline, no difference in Rap1-GTP levels was evident between the two cell lines (Fig. 5*A*). Previously, MAGI-1 was shown to be required for Rap-1 activation after the initiation of cell-cell contact in endothelial cells (11). To investigate this process in podocytes, we analyzed levels of GTP-Rap1 in confluent podocytes that had their intercellular contacts disrupted and then reestablished using a protocol adapted from Ref. 11. To accomplish this, we performed calcium switch experiments, where intercellular contacts were first disrupted by calcium chelation and then reestablished by restoring calcium-containing medium. We found that Rap1-GTP levels after calcium switch were significantly suppressed in *magi1* knockdown podocytes compared with controls (Fig. 5*B*). We next tested whether disrupted Rap1 signaling might be a potential mechanism for podocyte dysfunction in our bigenic mouse model. To do this, we combined glomerular lysates from two mice of the same genotype and then assayed for levels of Rap1-GTP. Whereas glomerular lysates from aged *magi1* knock-out mice showed only modestly diminished Rap1-GTP compared with nephrin heterozygous mice, there was a marked loss of glomerular Rap1-GTP in *magi1* knock-out mice that were also heterozygous for nephrin (Fig. 5*C*). This experiment was performed a total of three times (total of six mice per genotype group analyzed (18 total), three blots) with a representative image shown (Fig. 5*C*, *top*). Relative band intensity was quantified for each of the three experiments using LI-CORE Image Studios, composite ratios of active to total Rap1 were calculated for each blot, and statistical analysis was performed (Fig. 5*C*, *bottom*). These data suggested that over time, MAGI-1 and nephrin function synergistically to maintain activated Rap1. To look at this interaction in more detail, we overexpressed nephrin and MAGI-1 in a conditionally immortalized human podocyte cell line and then analyzed levels of GTP-Rap1 in the setting of calcium switch (Fig. 5*D*). We found that the combined overexpression of nephrin and MAGI-1 augmented levels of Rap-GTP in response to calcium switch. Importantly, the introduction of a mutant MAGI-1 that is missing part of its nephrin-interacting domain (PDZ domain 3 (2)) abrogated this effect, suggesting that a direct interaction between MAGI-1 and nephrin is required to facilitate signal transduction.

## Discussion

Vertebrates express three distinct MAGI scaffolds: MAGI-1, MAGI-2, and MAGI-3. Recent data clearly demonstrate MAGI-2 to be the dominant renal MAGI isoform, as reflected by the severe podocyte phenotype of *magi2* null mice (5–7) without functional compensation by the expression of MAGI-1. These studies suggest that MAGI-2, via its multiple protein-interacting domains, functions to assemble and maintain the structural integrity of the slit diaphragm complex. Nephrin, for example, is dramatically mislocalized in knock-out mice lacking MAGI-2 (7). Our studies suggest that MAGI-1, on the other hand, does not have a role in slit diaphragm assembly, and under basal conditions does not play a major role in stabilizing the complex over time. This is supported by long term normal glomerular architecture and function in *magi1* knock-out mice, including proper localization of nephrin and neph1. This suggests that at baseline, the absence of MAGI-1 can be compensated for by expression of other genes.

We hypothesized that a second mild but complementary genetic insult could enhance susceptibility to glomerular damage in *magi1* knock-out mice. Such a “multihit” scenario is typical for glomerular disease pathogenesis, including human FSGS, where two or more insults may be required for disease initiation (17–19). To identify candidate genes likely to provide such a “second hit,” we utilized the developing fly eye as a model to test for functional complementation between slit diaphragm molecules and MAGI. We found that diminished expression of either Hibris or Roughest (14, 15) could dramatically exacerbate the genetic effects of MAGI depletion. These results provided a rationale for analyzing the effects of nephrin and neph1 haploinsufficiency in our *magi1* knock-out mouse model. One clear limitation of our approach is that because MAGI is the sole fly homolog of the three mammalian MAGI genes, it is difficult to ascertain whether its function correlates most closely with mammalian MAGI-1 or MAGI-2, the predominant MAGI isoforms expressed at the slit diaphragm. Available evidence suggests correlation with MAGI-1 to be more likely. Whereas null mutations of fly Hibris (20) and Roughest (21–23) each induce severe dysfunction of eye development that closely correlate with the severe podocyte phenotypes of mammalian nephrin and neph1 loss of function mutants, *magi* null mutant flies demonstrate only a subtle eye phenotype with mild roughness (24). This is more in line with the mild phenotype evident in our *magi1* null mouse line. Further testing would be required to confirm this conclusion.

Our mouse data suggest that nephrin heterozygosity, which fails to induce proteinuria on its own (9), is capable of providing podocytes with a second hit when combined with complete loss of MAGI-1, resulting in spontaneous FSGS. The same does not appear to be true for neph1 heterozygosity, which fails to induce proteinuria on its own (10) and does not induce spontaneous FSGS when combined with loss of MAGI-1. Even with the bigenic addition of nephrin heterozygosity, the observed FSGS phenotype in *magi1* null mice takes many months to manifest and does so with a low penetrance, suggesting that the combined genetic effects on podocyte function are still relatively mild and require additional factors for FSGS initiation. One such factor could be the loss of podocyte number and density that occurs as a part of normal aging (25). This could potentially accelerate the podocyte injury response over time and explain the late onset and low penetrance of FSGS in our model. Another explanation for the late onset phenotype is that experiments were done on a C57Bl/6 genetic background, which is known to be resistant to all types of kidney injury, including those of the glomerulus. It is possible that conducting identical experiments in a more susceptible background would have yielded a more robust podocyte phenotype. Current concepts of glomerular filter homeostasis emphasize the need for integrated functions of the various components of the capillary wall (slit diaphragm, glomerular basement membrane, and endothelial cell) (26). Despite the relative mildness of the mouse phenotype, MAGI-1, in concert with nephrin and probably other slit diaphragm proteins, probably plays a role in this process.

Although the down-regulation of nephrin expression in glomerular diseases has long been known, that nephrin deficiency in adult mice was a genetic risk factor for podocyte injury has only recently been clearly established (27). Using an inducible RNA interference-mediated nephrin knockdown mouse model, Li *et al.* (27) have shown that acquired loss of nephrin in adult mice, ∼80% knockdown, was associated with glomerular pathological changes with mild proteinuria after 20 weeks of persistent nephrin knockdown as well as increased susceptibility to glomerular injury. In human patients as well, steroid-resistant nephrotic syndrome cases have clearly been linked to complex heterozygous mutations of nephrin in both children and adults (19, 28–31), with a combined heterozygous mutation in WT1 being the best described. These findings clearly suggest that nephrin haploinsufficiency could be a genetic risk factor for FSGS when accompanied by other appropriate genetic and/or environmental risks. The consequences of reduced glomerular neph1 expression in adult mice have not been reported, and there are no reported cases of monogenic or bigenic neph1 mutations leading to steroid-resistant FSGS in humans. In mice, combined heterozygosity of neph1 and CD2AP did not induce FSGS, as opposed to bigenic heterozygosity of CD2AP with synaptopodin or Fyn (17). Our results similarly show that a reduced neph1 gene dose cannot induce proteinuria in *magi1* null mice.

**FIGURE 6.**
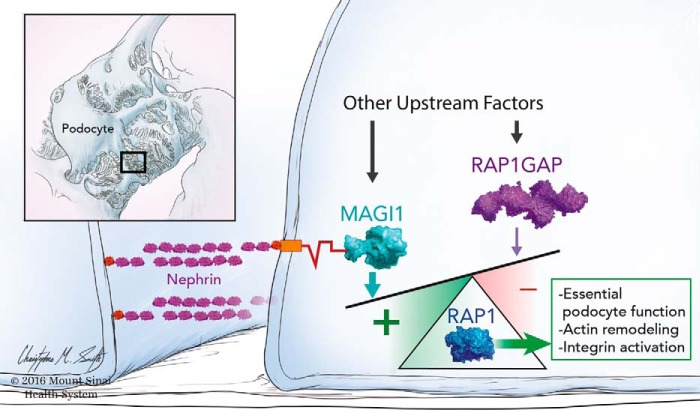
**Nephrin·MAGI-1 complex sustains podocyte Rap1 signaling.** In normal podocytes, multiple upstream factors, including both positive (GEFs) and negative (GAPs), work in concert to maintain proper Rap1 downstream signaling, including integrin activation and actin cytoskeletal remodeling. In the current work, we demonstrate that the interaction between MAGI-1 and nephrin enhances the accumulation of activated Rap1 under the basal condition. This positive drive is essential for maintaining long term slit diaphragm function as is reflected by the spontaneous FSGS phenotype evident in *magi1* KO mice that are also haploinsufficient in nephrin. This signal may counteract other negative factors, such as Rap1GAP accumulation, which we previously reported as an important factor that drives podocyte injury in FSGS (12).

Our *in vivo* studies from both flies and mice clearly demonstrate genetic complementation between MAGI-1 and nephrin, but the mechanism by which this interaction affects long term podocyte function was not known. The normal localization of slit diaphragm proteins, including nephrin, in our FSGS mouse model, does not support the notion that scaffolding loss was primarily responsible for podocyte dysfunction. Because MAGI-1 is a known upstream regulator of Rap1 (11), a small GTPase whose activation must be tightly balanced to sustain proper podocyte function (12), we hypothesized that changes in podocyte Rap1 signaling may instead be responsible. Our data do show that loss of MAGI-1 diminishes levels of podocyte Rap1 activation over time, but in a way that is largely dependent on its interaction with nephrin, the major outside-in signaling hub of the podocyte (Fig. 6) (8). Our overall understanding of the signals that are transmitted by the nephrin molecular complex are only beginning to be understood, but it is clear that tyrosine phosphorylation events link the nephrin complex to actin polymerization and remodeling (8). Although our cell culture data suggests that a direct interaction between nephrin and MAGI-1 is required, the exact mechanism of Rap1 activation by the complex is uncertain and probably requires other intermediary molecules, including a probable direct interaction between MAGI-1 and an unidentified guanine nucleotide exchange factor. Ongoing studies will be required to determine whether tyrosine phosphorylation of nephrin is required for Rap1 activation signaling. Furthermore, any diminution of podocyte Rap1 activation levels in the setting of MAGI-1 loss could be compensated for by changes in other positive and negative upstream regulatory elements, including potentially MAGI-2. This may partially explain the late onset and low penetrance of our observed mouse phenotype. We cannot exclude the possibility that other signaling pathways may also play a pathogenic role in our FSGS model.

We acknowledge the need to definitively correlate our findings with human FSGS pathogenesis. Our group is actively looking for convincing human FSGS cases with combined MAGI-1 and nephrin mutations; however, until now, we have not been able to clearly identify such a patient. Despite this, we continue to recommend including MAGI-1 sequencing in any comprehensive genetic testing for familial FSGS, particularly in those cases with clear heterozygous mutations in nephrin.

Overall, our work elucidates a novel interaction that plays an important role in Rap1 signal transduction from the slit diaphragm. We speculate that a similar mechanism may also contribute to the pathogenesis of human glomerular diseases, including FSGS.

## Experimental Procedures

### 

#### 

##### Drosophila Studies

The *magi RNAi* transgene (*magi^RNAi^*) was combined with either *rst RNAi* (*rst^RNAi^*), *hbs RNAi* (*hbs^RNAi^*), or *GFP*. Males of the flies carrying these transgenes were crossed with GMR-Gal4 flies. Eyes of the female pupae with the following genotypes were dissected at 40 h after puparium formation (APF): 1) *GMR-Gal4*/+; *UAS-GFP*/+; 2) *GMR-Gal4/UAS-magi^RNAi^*; *UAS-GFP*/+; 3) *UAS-rst^RNAi^*/+; *GMR-Gal4*/+; *UAS-GFP*/+; 4) *UAS-hbs^RNAi^*/+; *GMR-Gal4*/+; *UAS-GFP*/+; 5) *UAS-rst^RNAi^*/+; *GMR-Gal4/UAS-magi^RNAi^*; 6) *UAS-hbs^RNAi^*/+; *GMR-Gal4/UAS-magi^RNAi^*.

The *magi^RNAi^* transgenic flies were purchased from the Vienna *Drosophila* Resource Center (Vienna, Austria). The *rst^RNAi^* and *hbs^RNAi^* lines were described previously (14). *GMR-Gal4* and *UAS-GFP* were obtained from the Bloomington *Drosophila* Stock Center (Bloomington, IN).

Immunostaining of the pupal eye was performed as described (14). Rat anti-DE-cadherin (1:20) was obtained from the Developmental Studies Hybridoma Bank at the University of Iowa. Secondary antibodies used were as follows: Alexa 488- and Alexa 568-conjugated secondary antibodies (1: 5000; Molecular Probes); Cy5-conjugated secondary antibodies (1:1000; Jackson ImmunoResearch Laboratories). Images were captured using either an Axioplan2 epifluorescence microscope equipped with an Axiocam digital camera (Carl Zeiss, Inc.) or a TCS SP5 confocal laser-scanning microscope (Leica Microsystems Inc.).

Quantification of defects in the pupal eyes was carried out as described (32). A total of 75 hexagonal fields were analyzed for each genotype.

##### Generation of magi1 Global Knock-out Mice

*magi1* null mice were generated by homologous recombination (inGenious Targeting Laboratory, Inc., Ronkonoma, NY). Mice were back-crossed 10 generations onto a C57Bl/6 genetic background. Mice were maintained and sacrificed based on protocols approved by the Icahn School of Medicine at Mount Sinai institutional animal care and use committee. Genotyping was performed using the following primer pairs: S1, 5′-GAGCCCGGAAAGTTTGTTTT-3′; A1, 5′-GCCAGTCCAGTGGTTCTTCT-3′; S2, 5′-ATTAAGGGCCAGCTCATTCC; A2, 5′-TGACTCCCAGCACGTCATAG.

##### Nephrin and neph1 Mice

Nephrin knock-out mice were purchased from Jackson Laboratories and genotyped per their protocol. *Neph1* mice were developed by Tobias Huber (33) and genotyped using the following primer pairs: 5′-GAAAAGAGAGATACGGAGAACCGAGGG-3′ (forward, common), 5′-AGAACACTAAGGCAGCAAAAGAGAAGACGA-3′ (reverse, wild type), and 5′-CACAAAGCTGAGAAGAAAGGAAACCGT-3′ (reverse, knock-out). Expected product lengths are 750 bp for wild type allele and 600 bp for knock-out allele.

##### Antibodies and Plasmids

The following antibodies were used: nephrin (courtesy of Lawrence Holzman, University of Pennsylvania School of Medicine), synaptopodin (mAb G1, courtesy of Peter Mundel, Harvard University), MAGI-1 (Western blotting, Sigma-Aldrich, M5691), MAGI-1 (immunofluorescence, Santa Cruz Biotechnology, Inc., sc100326), MAGI-2 (Sigma-Aldrich, SAB4503718), FLAG (mAb M2, Sigma-Aldrich), rap1 (Millipore, 17-321), c-Myc-agarose (Sigma-Aldrich), and vimentin (Millipore, ABI1620).

The expression plasmids neph1-FLAG and nephrin-FLAG were courtesy of Puneet Garg (University of Michigan) and Peter Chuang (Icahn School of Medicine at Mount Sinai), respectively. MAGI-1 expression plasmids were described previously (16, 34). The Myc-sdk-1Δ expression plasmid encodes a C-terminal deletion mutant of the adhesion molecule sidekick-1 (16), which was used as a negative control for co-immunoprecipitation experiments.

##### Histopathology and Immunofluorescence

All kidneys were perfused *in vivo* with 4% paraformaldehyde. For histopathology, kidneys were left in paraformaldehyde overnight and then embedded in paraffin. Sections were cut at 2-μm thickness and then stained with PAS. For immunofluorescence, kidneys were fixed in PFA for 4 h, transferred to 18% sucrose overnight, and then flash-frozen in OCT medium. For electron microscopy, samples were cut into 1-mm cubes and fixed overnight in 2.5% glutaraldehyde before being epoxy-embedded using standard techniques (JEOL 1011 electron microscope).

##### Podocyte Cell Culture

The generation and propagation of a well established human podocyte cell line was described previously (35). Transient transfections were performed using Lipofectamine 3000 (Invitrogen) or electroporation (Amaxa Nucleofecter Kit; Lonza) according to the manufacturer's instructions.

##### Lentiviral Production and Infection

For *magi1* knockdown, lentiviral expression vectors carrying shRNAs were purchased from Sigma-Aldrich (Mission shRNA). Infections were done at the permissive temperature in human podocytes, and then stable cell lines were established by selection with puromycin. Stable MAGI-1 knockdown lines were grown at 37 °C for at least 7 days before their use in experiments. All lentiviral preparation and infections were performed as described previously (36).

##### RNA Extraction and Quantitiative PCR

RNA was extracted from the kidney cortex using the RNeasy purification kit(Qiagen). Quantitative PCR was performed at the Icahn School of Medicine at Mount Sinai Quantitative PCR Shared Research Facility. The facility uses an ABI PRISM 7900HT sequence detection system using SYBR Green. Primers were as follows: nephrin, 5′-GTGCCCTGAAGGACCCTACT-3′ (forward) and 5′-CCTGTGGATCCCTTTGACAT-3′ (reverse); neph1, 5′-CCTGGCCCTACTCGTTTTGAT-3′ (forward) and 5′-CTGTGCCATAGTCAGAGGCAG-3′ (reverse); GAPDH, 5′-GCCATCAACGACCCCTTCAT-3′ (forward) and 5′-ATGATGACCCGTTTGGCTCC-3′ (reverse).

##### Calcium Switch

Confluent human podocytes were serum-deprived overnight in medium containing 1% serum. Cells were exposed to 4 mm EGTA for 30 min to chelate extracellular calcium and disrupt intercellular contacts (11). Cells were washed twice with 1× PBS and then returned to full serum calcium-containing medium for the indicated time.

##### Co-immunoprecipitations

293T cells were doubly transfected with Myc-MAGI-1 and either FLAG-tagged nephrin, neph1, or sdk-1Δ. Total cellular lysates from each were then incubated with Myc beads (Clontech) overnight at 4 °C. After washing beads three times and boiling, Western blotting was performed using an anti-FLAG M2 antibody (Sigma-Aldrich).

##### Glomerular Isolation

Mouse glomeruli were isolated as described previously (37). Briefly, mice were perfused with HBSS containing 2.5 mg/ml iron oxide and 1% bovine serum albumin. After perfusion, the kidneys were removed, minced into 1-mm^3^ pieces, and then digested in HBSS containing 1 mg/ml collagenase A and 100 units/ml deoxyribonuclease I. Digested tissue was then passed through a 100-μm cell strainer and collected by centrifugation. The pellet was resuspended in HBSS, and glomeruli were collected using a magnet.

##### Adriamycin Nephropathy

Doxorubicin hydrochloride at a dose of 18 mg/kg was injected intravenously via tail vein into male mice. Mice were administered 2 ml of intraperitoneal normal saline once daily for the first 3 days after adriamycin injection. Urine was collected on days 7 and 14 and then assayed for protein/creatinine ratio.

##### Rap1-GTP Pull-downs

Relative levels of Rap1-GTP were assessed by pull-downs using a GST-tagged fusion protein, corresponding to amino acids 788–884 of the human RalGDS-RAP-binding domain bound to glutathione-agarose as per the manufacturer's instructions (Millipore, 17-321).

##### Measurement of Albumin Flux

After 3 days postconfluence, podocytes were washed with 1× PBS, and then the medium in the upper and lower chambers was replaced with serum-free medium. Texas Red-labeled BSA at a concentration of 50 μg/ml was added to the lower chamber. Aliquots of medium were removed from the upper chamber at regular time intervals, and the fluorescence (excitation 590 mm, emission 625 mm) was measured. A standard curve was generated from measurements of serial dilutions of Texas Red-labeled BSA to calculate the amount of BSA that had moved to the upper chamber.

##### Digital Image Analysis

Cells were labeled for nephrin-FLAG or neph1-FLAG. Images were acquired at the Mount Sinai Microscopy Core Facility using a Zeiss Axioplan fluorescence microscope. All parameters, including exposure time, were kept constant while acquiring the images. Images were analyzed using ImageJ software. Regions of interest were selected manually, specifically selecting the cell boundary region. Representative images from at least three separate experiments are given. At least 40 randomly selected cells from each experiment were included in the analysis.

## Author Contributions

S. B., R. I. J., A. C., and L. K. conceived the studies. J. N., S. B., R. I. J., B. Z., J. L., J. V., and L. K. conducted experiments and acquired the data. S. B., R. I. J., K. N. C., J. C. H., V. D. D., and L. K. analyzed the data. F. G., T. B. H., and A. C. provided materials. S. B., R. I. J., C. M. S., and L. K. wrote the manuscript with contributions from all coauthors. All authors reviewed the results and approved the final version of the manuscript.

## Supplementary Material

Supplemental Data

## Footnotes

3 The abbreviations used are:

GAP: GTPase-activating protein
GEF: guanine nucleotide exchange factor
IOC: interommatidial cell
FSGS: focal segmental glomerulosclerosis
PAS: periodic acid-Schiff
HBSS: Hanks' balanced salt solution.
