# Supplementary material for: MAGI-1 Interacts with Nephrin to Maintain Slit Diaphragm Structure through Enhanced Rap1 Activation in Podocytes
Source: J Biol Chem. 2016 Oct 5;291(47):24406–17. doi: 10.1074/jbc.M116.745026 (PMC5114397; doi:10.1074/jbc.M116.745026)
Supplement: Supplemental Data [file supp_291_47_24406__index.html]

MAGI-1 Interacts with Nephrin to Maintain Slit Diaphragm Structure Through Enhanced Rap1 Activation in Podocytes — MAGI-1 Interacts with Nephrin to Maintain Slit Diaphragm Structure through Enhanced Rap1 Activation in Podocytes — MAGI-1 Sustains Podocyte Rap1 Activation — Supplemental Data 

# MAGI-1 Interacts with Nephrin to Maintain Slit Diaphragm Structure through Enhanced Rap1 Activation in Podocytes

## Supplemental Data

- Quantification of pupal eye patterning defects (.pdf, 564 KB) - Supplementary table 1
