# Supplementary material for: MAGI-1 Interacts with Nephrin to Maintain Slit Diaphragm Structure through Enhanced Rap1 Activation in Podocytes
Source: J Biol Chem. 2016 Oct 5;291(47):24406–17. doi: 10.1074/jbc.M116.745026 (PMC5114397; doi:10.1074/jbc.M116.745026)
Supplement: Supplemental Data [file 10.1074_M116.745026_jbc.M116.745026-1.pdf]

Table 1: Quantification of pupal eye patterning defects

GENOTYPE	OMS [1.]			relevant p-values [2.]			
	**Mean**	**SD**	**SE**	*GMR >GFP*	*GMR >magi*^{RNAi}	*GMR >hbs*^{RNAi}	*GMR >rst*^{RNAi}
GMR >GFP	**0.45**	0.81	0.09	-	-	-	-
GMR >hbs^{RNAi}	**2.16**	1.90	0.22	1.3×10^{-10}	-	-	-
GMR >rst^{RNAi}	**2.22**	2.19	0.28	2.8×10^{-5}	-	-	-
GMR >magi^{RNAi}	**2.31**	1.76	0.20	3.9×10^{-13}	-	-	-
GMR >magi^{RNAi}*, hbs*^{RNAi}	**6.73**	2.86	0.33	-	4.74×10^{-21}	1.34×10^{-21}	-
GMR >magi^{RNAi}*, rst*^{RNAi}	**7.01**	2.84	0.33	-	5.29×10^{-23}	-	2.40×10^{-26}

Notes:

1. 75 hexagonal fields of each genotype were analyzed. The Ommatidial Mispatterning Score (OMS) is the mean number of errors observed in each hexagonal field; SD = standard deviation, SE = standard error.

2. Student T-tests compared total patterning errors in relevant datasets. P-values below 0.01 are statistically significant at the 1% confidence level.
